# Carbonated wollastonite – An effective supplementary cementitious material?

**DOI:** 10.1111/jmi.13067

**Published:** 2021-11-03

**Authors:** Andreas Leemann, Frank Winnefeld, Beat Münch, Florian Läng

**Affiliations:** ^1^ Empa, Swiss Federal Laboratories for Materials Science and Technology Dübendorf Switzerland; ^2^ Holcim (Schweiz) AG Würenlingen Switzerland

**Keywords:** carbonation, compressive strength, microstructure, supplementary cementitious material, wollastonite

## Abstract

Carbonated wollastonite clinker (CS) may be suitable as supplementary cementitious material (SCM) for mortar and concrete. The microstructure of unground CS clinker, carbonated CS slurry and a mortar blended with carbonated CS are investigated by scanning electron microscopy. Additionally, a reference mortar with pure Portland cement and one with a cement replacement level of 30 mass‐% by carbonated CS are produced to assess its contribution to compressive strength development. The calcium silicates are decalcified during carbonation resulting in CaCO_3_ and amorphous SiO_2_. The latter reacts when used as SCM in mortar influencing the Ca/Si ratio of calcium‐silicate‐hydrate and contributing to compressive strength development.

## 1 INTRODUCTION

A way of reducing CO_2_ emissions linked to the production of mortar and concrete is the substitution of cement clinker with supplementary cementitious materials (SCM). A potential candidate to expand the traditional range of SCM including fly ash, blast furnace slag, metakaolin and silica fume could be wollastonite (CaSiO_3_, CS according to cement notation). Because the reserves of natural wollastonite are relatively small (total of 0.100 × 10^9^ tons compared to 4.1 × 10^9^ tons yearly global cement production),^1^, ^2^ such deposits can be expected to cover mainly local demand. Only industrially manufactured wollastonite is of potential interest for the global cement industry. It can be produced in conventional cement kilns at a temperature of ∼1200°C resulting in a 30% lower CO_2_ emission compared to Portland cement clinker.^3^ While CS clinker is not reactive in the alkaline environment, it is prone to carbonation. In dry conditions, CaSiO_3_ carbonates very fast initially along its cleavage plane.^4^ CO_2_ reacts with the surface oxygen forming CO_3_^2−^ complexes. However, as soon as a carbonate monolayer has formed, the reaction essentially stops. A different type of mechanism takes place in the presence of liquid water. The metal–proton exchange reaction leads to the leaching of calcium from the surface of CS.^5^ The dissolved calcium reacts with CO_2_ and forms CaCO_3_.^6^ In addition to CaCO_3_, silica gel is formed.^7^ ^29^Si magic‐angle‐spinning nuclear magnetic resonance shows that the ‘silica gel’ is a Ca‐modified phase consisting mainly of Q^3^ and Q^4^ species with minor amounts of Q^2^ and Q^1^.^8^ The silica‐rich gel in carbonated CS may have the potential to participate in cement hydration.

In this project, the microstructure of the original CS clinker, the carbonated CS slurry and the hydration products formed in mortar blended with carbonated CS are studied in order to understand the relation between the phases present, their spatial distribution and the resulting reactivity. Scanning electron microscopy (SEM) in combination with energy‐dispersive X‐ray spectroscopy (EDS) is used for characterisation. Additionally, the compressive strength of a reference mortar and the mortar containing carbonated CS are determined.

## 2 MATERIALS AND METHODS

### 2.1 Materials

The chemical composition of the Swiss Portland cement (PC) CEM I 42.5 N and the industrially produced CS clinker are given in Table 1. Additionally, the phases present in the CS clinker are provided in Table 1. Besides (pseudo)wollastonite (CaSiO_3_), rankinite (Ca_3_Si_2_O_7_) and belite (Ca_2_SiO_4_) are present as additional calcium silicates.

**TABLE 1:** Chemical composition of cement and CS clinker including the mineralogy of the latter determined by quantitative X‐ray diffraction

Material	CEM I 42.5 *N*	CS clinker		CS clinker
Parameter^1^	Mass‐%	Mass‐%	Phase^4^	Mass‐%
CaO	63.42	44.12	Pseudowollastonite	22.4
SiO_2_	19.86	43.42	Rankinite	30.6
Al_2_O_3_	4.86	3.93	Belite (ß)	1.9
Fe_2_O_3_	2.86	1.86	Bredigite	0.3
MgO	1.55	1.59	Åkermanite‐Gehlenite	22.1
Na_2_O	0.15	0.18	Calcite	1.0
K_2_O	0.85	0.56	Quartz	4.3
P_2_O_5_	0.235	0.170	Cristobalite	1.9
TiO_2_	0.251	0.199	Amorphous	15.5
MnO	0.050	0.053		
Cr_2_O_3_	0.005	0.005		
SO_3_	3.18	1.02		
L.O.I.^3^)	2.54	2.68		
Total	99.82	99.79		
CO_2_ ^2^)	1.61	1.91		

^1^ Oxide compositions except CO_2_ determined by X‐ray fluorescence analyses using fused beads according to EN 196‐2.

^3^ Loss on ignition according to EN 196‐2.

^2^ X‐ray diffraction (XRD) was performed using a PANalytical X'Pert Pro MPD diffractometer in a ϴ–ϴ configuration using CoKα radiation with a fixed divergence slit size of 0.5° and a rotating sample stage. The samples were scanned between 5° and 90° 2ϴ with the X'Celerator detector. The Rietveld refinement for phase quantification was performed with X'Pert High Score Plus V. 4.9 following the procedure and using the crystal structures recommended by Ref. (9). For pseudowollastonite, åkermanite‐gehlenite and cristobalite, the crystal structures reported in Refs. (10)–(12), respectively, were used. The amorphous content was quantified using the G‐factor method with CaF_2_ as external standard.^13^, ^14^

^4^ Determined as total carbon by combustion analysis according to ISO 10694 and recalculated to CO_2_.

The industrially ground clinker had a Blaine value of 7860 cm^2^/g and a density of 2.84 g/cm^3^. A slurry with a water‐to‐powder ratio (*w*/*p*) of 0.50 was produced and carbonated in a desiccator at 60°C, 100% CO_2_ and close to 100% relative humidity. The slurry solidified during carbonation and was briefly ground afterwards resulting in a Blaine value of 7760 cm^2^/g (density: 2.56 g/cm^3^). CaCO_3_ content after carbonation as derived by mass loss in thermogravimetric analysis (Mettler Toledo TGA/SDTA 851e) was 42 mass‐%.

Two mortar mixes were produced. The reference mortar (mortar REF) was produced with CEM I 42.5 N according to EN 196‐1 (quartz sand 0/2 mm, *w*/*p* = 0.50). In the second mortar (mortar CS), CEM I was partially preplaced with carbonated CS resulting in a mass ratio of 70/30. The mortar bars (40 mm × 40 mm × 160 mm) were demoulded after 24 h and stored in water until testing.

The uncarbonated CS clinker used for SEM analysis was not ground. It was embedded in epoxy resin, polished and carbon coated. Samples of the solidified carbonated CS clinker and the mortar used for scanning electron microscopy were dried at 50°C, impregnated with epoxy, polished and carbon coated.

### 2.2 Methods

A FEI Quanta 650 was used in the back‐scattering mode at a pressure of 3.5–5.0 × 10^−6^ Torr to acquire images. Energy‐dispersive X‐ray spectroscopy (EDS) was conducted with a Thermo Noran Ultra Dry 60 mm^2^ detector and Pathfinder X‐Ray Microanalysis Software, Version 2.4, applying phi‐rho‐Z corrections. Acceleration voltage for EDS point analysis and line scans was 12 kV at a spot size of 4.5 using K‐lines. Phase clustering based on element maps (acquired with an acceleration voltage of 20 kV at a spot size of 5 with a dead time of 35–45%) was performed according to Ref. (15). This procedure is based on a first clustering step, followed by an automated identification of the chemical compositions and a final clustering step applying a minimum Euclidean distance classifier. Emission current for analysis ranged from 130 to 140 μA.

As the grain size of clinker hydrates in the paste is much smaller than the interaction volume below the point of beam incidence, the EDS spectrum is generated by several phases in un‐known and variable proportions. Therefore, the results from EDS point analysis and line scans are presented as elemental ratio plots based on atom‐%. This is a well‐established approach in the EDS analysis of cementitious materials.^16^ Information on X‐ray diffraction (XRD) is given in Table 1.

Compressive strength was determined at the age of 2, 7, 28 and 90 days according to EN 196‐1.

## 3 RESULTS

### 3.1 CS clinker

The diameter of the unground CS clinker balls from the kiln in their initial state is usually between 5 and 15 mm. The presence of different phases in the clinker particles is indicated by differences in the backscattering contrast. EDS point analysis confirms the presence of wollastonite (Ca/Si ∼0.9), rankinite (Ca/Si ∼1.4) and belite (Ca/Si ∼1.85). Ca/Si ratio of gehlenite‐åkermanite ranges from 1.0 to 1.5 with varying contents of Mg and Al leading to a (Mg+Al)/Si ratio of 0.6–1.0. A phase with Ca/Si ∼0.9 and (Mg+Al)/Si ∼0.4 cannot be attributed to one of the phases given in Table 1. The phase with the lowest backscattering contrast is amorphous SiO_2_ with traces of Na, K and Ca. Additionally, minor amounts of CaCO_3_ and pure SiO_2_ interpreted as quartz (higher backscattering contrast compared to amorphous SiO_2_) and traces of CaSO_4_ are present.

**FIGURE 1:**
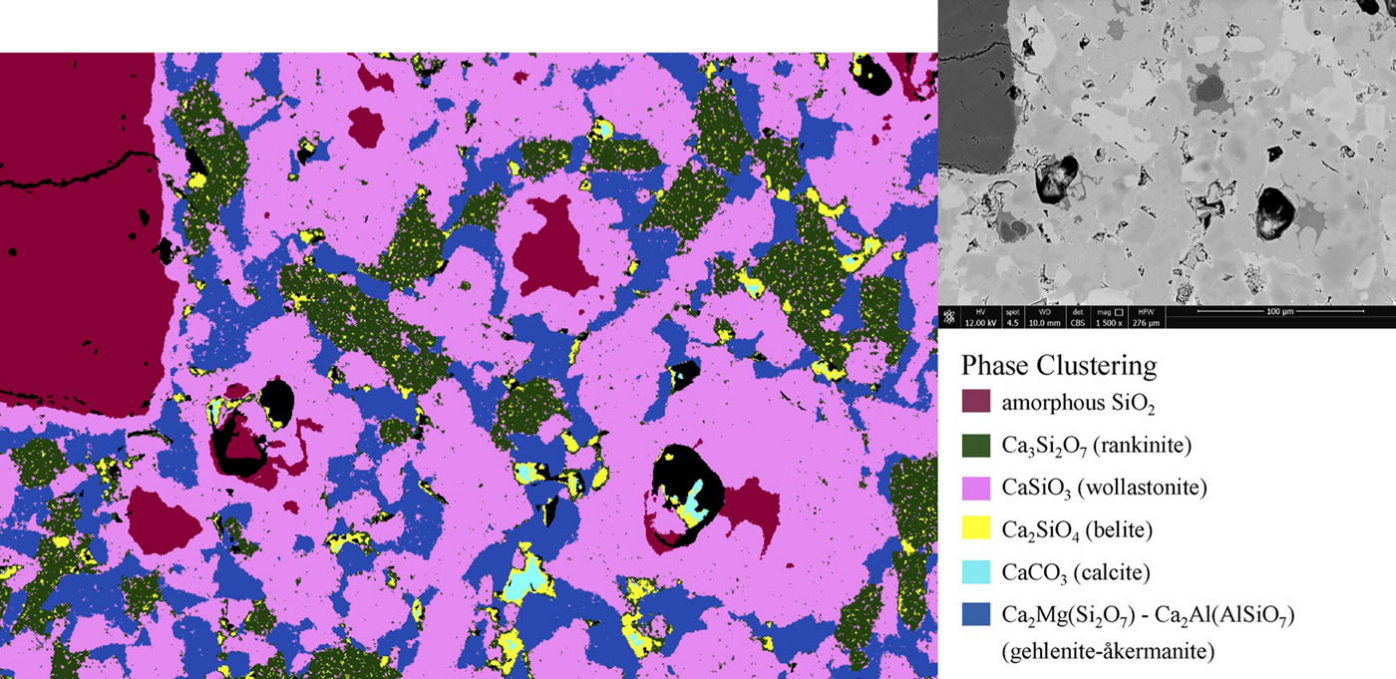
BSE image (upper right) and phase clustering based on element maps for the different phases present in a CS clinker particle. Horizontal field width (HFW) = 247 μm

The phase clustering based on element maps shows the spatial distribution of the different phases (Figure 1). Gehlenite‐åkermanite is present as interstitial phase. Belite occurs as finely distributed phase in clusters of rankinite or in immediate vicinity of CaCO_3_. Amorphous SiO_2_ is always enclosed by wollastonite. Although there are variations in the relative amounts of the various clinker phases from clinker particle to clinker particle, the texture of different clinker particles is usually similar.

### 3.2 Carbonated wollastonite

**FIGURE 2:**
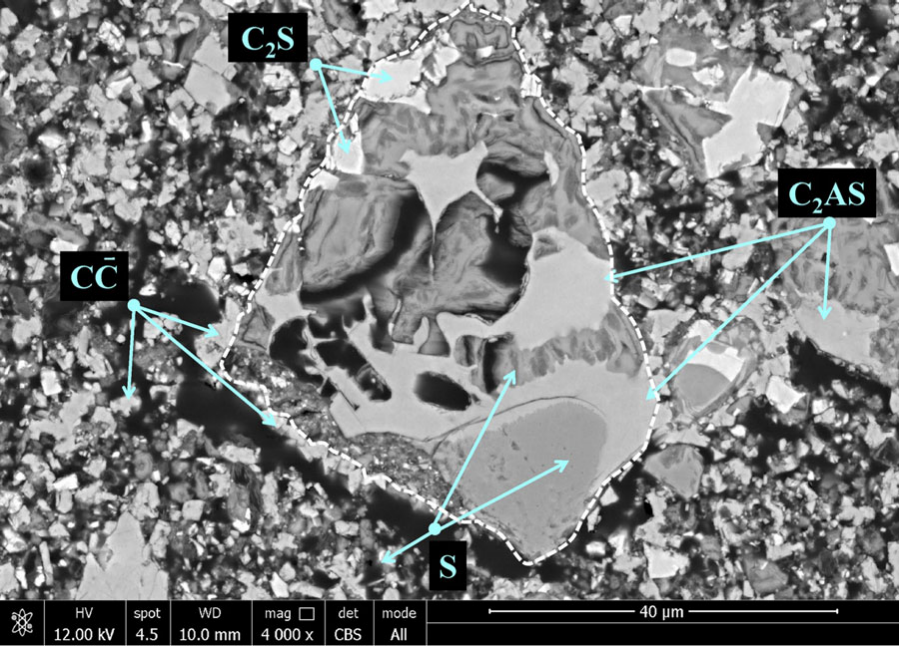
Solidified CS slurry after carbonation with amorphous silica (S), CaCO_3_ (CC), unreacted gehlenite‐åkermanite (C_2_AS‐C_2_MS_2_ solid solution) and belite (C_2_S). The white dotted line outlines the original form of the uncarbonated CS‐particle. Composition of phases given in cement notation. HFW = 103 μm

The slurry solidfied by carbonation contains gehlenite‐åkermanite, traces of uncarbonated calcium silicates, CaCO_3_ and amorphous silica (Figure 2). The amorphous silica contains some calcium leading to a Si/Ca ratio in the range of 4–50. This agrees well with the NMR results indicating the incorporation of Ca in amorphous SiO_2_.^6^ The amorphous silica present in the uncarbonated clinker was not identifiable anymore.

CaCO_3_ and amorphous silica are intermixed as separate fine particles <10 μm forming a porous assemblage in which larger particles (>30 μm) are embedded. Such larger particles may exhibit a partial fringe of CaCO_3_ adjacent to amorphous silica.

### 3.3 Mortar

**FIGURE 3:**
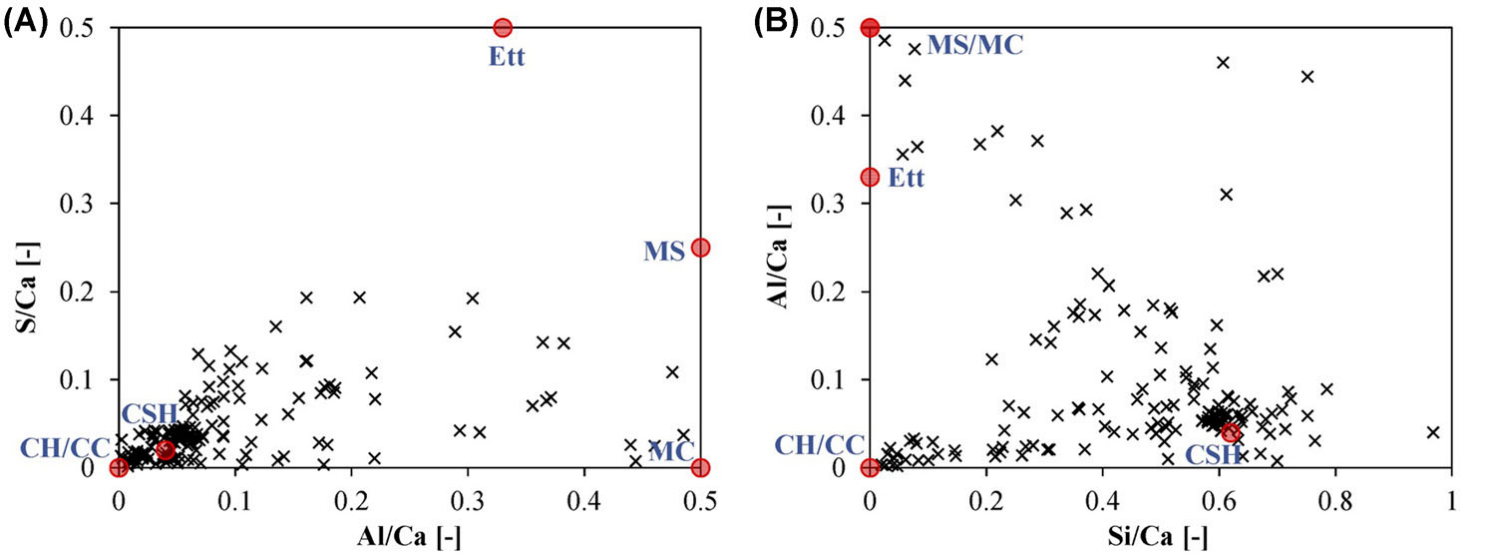
Atomic S/Ca ratio as a function of atomic Al/Ca ratio (A) and atomic Al/Ca ratio as a function of atomic Si/Ca ratio (B). The pure phase are indicated with the red dots (CH = portlandite, CC = CaCO_3_, CSH = calcium‐silicate‐hydrate, Ett = ettringite, MS = monosulfate, MC = monocarbonate)

The composition of the hydrates in the mortar at the age of 28 days is assessed with EDS line scans in the cement paste. The shifts of the analysed points from C‐S‐H towards higher S/Ca‐ and Al/Ca ratio indicate the presence of ettringite, monosulfate and monocarbonate (Figure 3A). CaCO_3_ contained in the carbonated wollastonite clinker leads to a relative high number of points shifting from C‐S‐H towards calcite‐portlandite in the Al/Ca to Si/Ca ratio plot compared to a pure PC system (Figure 3B). Additionally, it stands out that C‐S‐H exhibits an unusual high Si/Ca ratio of about 0.62. The value for a pure PC system determined with this type of analysis is typically about 0.54 and the one of a system with 65 mass‐% of slag (CEM III/B) 0.70.^17^ Amorphous SiO_2_ present in larger particles of carbonated CS can exhibit a reaction rim. The Si/Ca ratio in such rims ranges from 0.7 to 3.5.

### 3.4 Compressive strength

At the age of 2 days, the mortar with carbonated CS displays a considerable lower strength than the reference mortar with a value of 14.2 compared to 24.1 MPa. However, the difference is decreasing with increasing age resulting in values of 31.2 and 39.3 MPa at 7 days, followed by 47.5 and 51.5 MPa at 28 days. At 90 days, the compressive strength of both mortars is nearly equal at 55.8 and 56.6 MPa, respectively.

## 4 DISCUSSION AND SUMMARY

The spatial arrangement of the clinker phases within unground clinker particles typically shows a dependence on Ca content. Amorphous silica is enclosed by wollastonite, as the calcium silicate with the lowest Ca content. On the other hand, belite as the calcium silicate with the highest Ca content is spatially linked either to rankinite or calcite but not to wollastonite. Gehlenite‐åkermanite fills the interstitial space between these phases.

In the carbonated CS slurry, the porous assemblage formed by CaCO_3_ and amorphous silica and the CaCO_3_ rims occurring alongside amorphous silica in larger grains shows that the calcium silicates are decalcified during carbonation. Ca is dissolved and precipitates close to the original calcium silicate particle. The observed incorporation of a minor amount of Ca in the silica gel agrees well with NMR results.^6^ The porous assemblage of CaCO_3_ and amorphous SiO_2_ particles can easily be fractured by brief grinding. This makes the amorphous silica accessible for reaction when exposed to alkaline solutions. Gehlenite‐åkermanite is resistant to CO_2_‐uptake.

The fact that the amorphous silica present in the uncarbonated clinker is not identifiable anymore after carbonation, suggests that it is transformed as well. It can be assumed that the alkalis present before carbonation have been dissolved in the slurry and carbonated. Additionally, the calcium dissolved during the carbonation process has likely led to Ca uptake of the amorphous silica like it is typically observed in cementitious systems.^18^ This makes it indistinguishable from the amorphous silica formed during carbonation.

The presence of AFm is clearly indicated (Figure 3), but it is likely that no monosulfate but only monocarbonate is formed, as it is typical for hydrating PC systems containing limestone.^19^

In mortar CS, the relatively high Si/Ca ratio of C‐S‐H with a value of 0.62 is between the one of a pure PC system (0.54) and the one of a system with 65 mass‐% of slag (0.70).^8^ The Si/Ca ratio and the reactions rims occasionally observed in larger particles clearly show that the amorphous SiO_2_ of carbonated CS participates in cement hydration. This is confirmed by the results of compressive strength testing.

The diminishing difference in relative compressive strength of mortar CS to mortar REF with increasing age from 41.1 (2 days), 20.6 (7 days), 7.8 (28 days) to 1.4% (90 days) indicates that the contribution to strength development and the kinetics of reaction of carbonated CS is superior compared to a low CaO‐fly ash.^20^

## 5 OUTLOOK

Carbonated CS looks like a possible candidate to extend the range of existing SCM. However, the durability aspects have to be investigated for a further assessment. Additionally, life cycle analysis taking into account the entire production and distribution chain of carbonated CS should reveal, if it really is a sensible candidate when sustainability is considered.
